# Synchronous thyroid cancer and malignant struma ovarii: concordant mutations and microRNA profile, discordant loss of heterozygosity loci

**DOI:** 10.1186/s13000-023-01336-6

**Published:** 2023-04-18

**Authors:** Gabriella T. Seo, Jeremy Minkowitz, Danielle A. Kapustin, Jun Fan, Gerald Minkowitz, Miriam Minkowitz, Eric Dowling, Ammar Matloob, Divya Asti, Meekoo Dhar, Christopher Shutty, Alan Brickman, Mark L. Urken, Margaret Brandwein-Weber, Sydney D. Finkelstein

**Affiliations:** 1 Thyroid, Head and Neck Cancer (THANC) Foundation, 10 Union Square East, Suite 5B, New York, NY 10003 USA; 2 Minkowitz Pathology, 904 49th Street, Brooklyn, NY 11219 USA; 3 SUNY Downstate Medical Center, 450 Clarkson Avenue, Brooklyn, NY 11203 USA; 4 Department of Otolaryngology-Head and Neck Surgery, Icahn School of Medicine at Mount Sinai, 10 Union Square East, Suite 5B, New York, NY 10003 USA; 5 Department of Pathology, Mount Sinai West Medical Center, 1000 10th Avenue, New York, NY 10019 USA; 6 Department of Hematology and Medical Oncology, Northwell Health Staten Island University Hospital, 475 Seaview Avenue, Staten Island, NY 10305 USA; 7 ParCare Community Health Network, 6010 Bay Parkway, Brooklyn, NY 11204 USA; 8 Interpace Diagnostics, 2515 Liberty Ave, Pittsburgh, PA 15222 USA

**Keywords:** Malignant struma ovarii, Papillary thyroid cancer, Loss of heterozygosity, microRNA

## Abstract

**Background:**

Struma ovarii is an unusual ovarian teratoma containing predominantly thyroid tissue. Less than 10% of cases undergo malignant transformation in the thyroid tissue and are considered malignant struma ovarii (MSO). MSO have been reported with concurrent thyroid lesions, but molecular data is lacking.

**Case presentation:**

A 42-year-old female developed MSO and synchronous multifocal subcentimeter papillary thyroid carcinoma (PTC). The patient underwent a salpingo-oophrectomy, thyroidectomy, and low-dose radioactive iodine ablation. Both the thyroid subcentimeter PTC and MSO were positive for BRAF V600E mutation, and microRNA expression profiles were similar across all tumor deposits. However, only the malignant component demonstrated extensive loss of heterozygosity (LOH) involving multiple tumor suppressor gene (TSG) chromosomal loci.

**Conclusions:**

We present the first reported case of MSO with synchronous multifocal subcentimeter PTC in the thyroid containing concordant BRAF V600E mutations and resulting with discordant LOH findings. This data suggests that loss of expression in tumor suppressor gene(s) may be an important contributor to phenotypic expression of malignancy.

## Background

Struma ovarii is an unusual type of mature ovarian teratoma comprised predominantly of thyroid tissue (at least 50%) [1]. These tumors account for about 1% of ovarian neoplasms [2], and fewer than 10% undergo malignant transformation [3]. A small portion of patients with malignant struma ovarii (MSO) have synchronous primary thyroid carcinoma [4]. In such rare cases, ovarian surgery is indicated; however, synchronous thyroid management remains disputed. Management decisions are further complicated by the difficulty of distinguishing between synchronous independent malignancies versus a single malignancy with metastasis and molecular data is lacking.

In the present study, we examine current literature and present a unique case of MSO with synchronous primary thyroid carcinoma. Tumor relatedness was defined using three approaches: mutational profiles, pattern of microRNA (miRNA) expression, and the presence and extent of tumor suppressor gene (TSG) loss. Our findings shed light on the pathobiology of thyroid subcentimeter papillary thyroid carcinoma (PTC) and its differentiation from usual forms of PTC. We describe a potential causal role of acquired TSG loss in the development of PTC. Our study suggests the possibility of additional genetic changes associated with PTC which may be used as diagnostic tools upon further validation.

## Case presentation

**Fig. 1:**
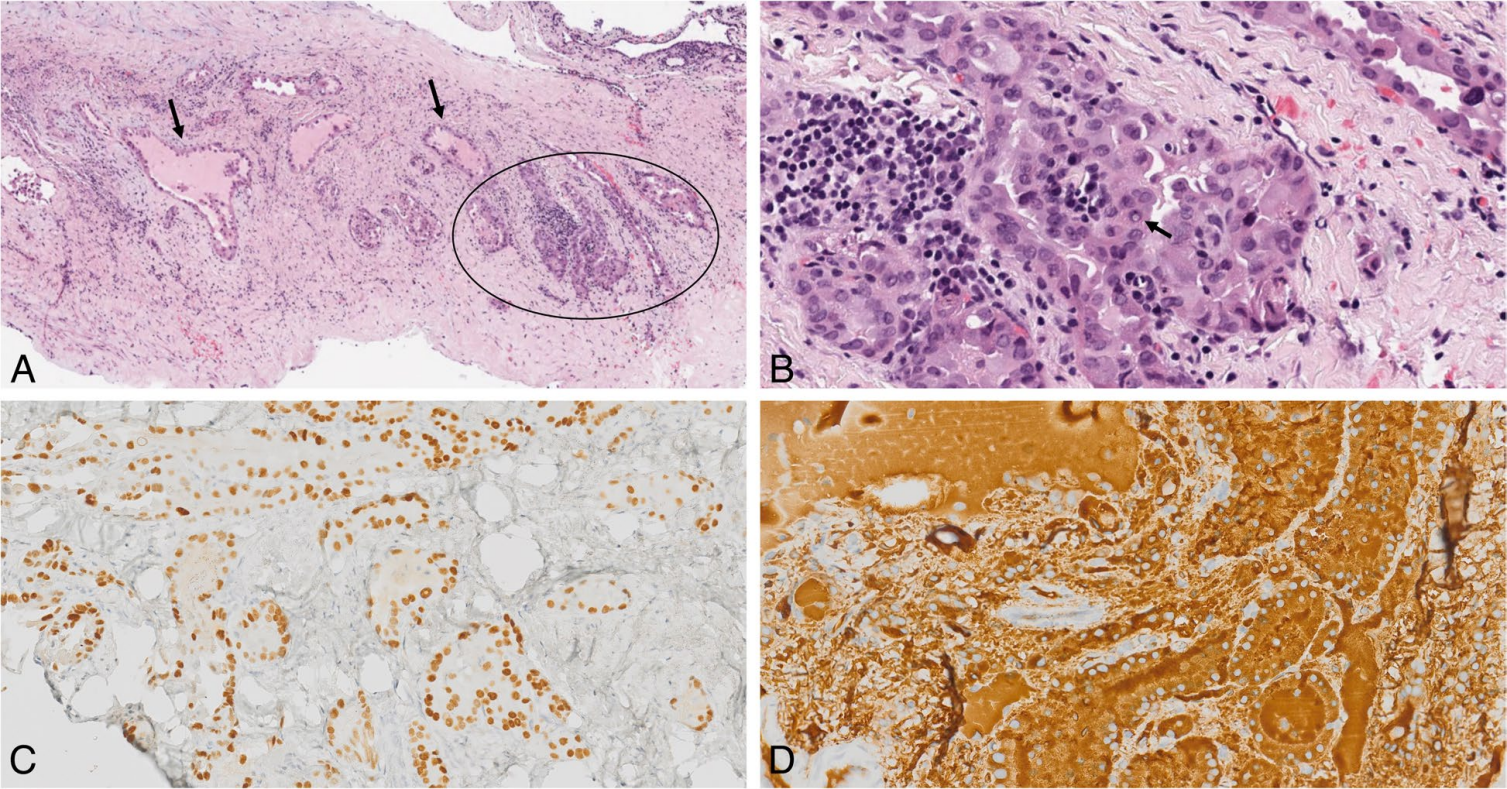
Malignant Struma Ovarii. **A** Low power view (0.4x) of H&E section from the left ovary shows thyroid follicles of various sizes and shapes embedded in an inflamed fibrotic stroma. Note the malignant aggregate of thyroid tissue (circled) and adjacent poorly formed thyroid follicles (arrow). **B** High power image (20x) of the malignant component with nuclear pleomorphism, nuclear overlapping, irregular nuclear contour, and nuclear pseudoinclusions (arrow). These cells are positive for TTF-1 (**C**) and thyroglobulin (**D**). Magnification in panel **C** and **D** is demonstrated by a scale bar

A 42-year-old female presented with left-sided pelvic pain. Imaging revealed an enlarged left ovary measuring 10.5 × 5.4 × 8.6 cm. The patient underwent a left salpingo-oophrectomy. The gross specimen arrived fragmented measuring 9.5 cm in greatest dimension. The specimen was entirely submitted in 29 slides. Histologic sections demonstrated struma ovarii with three foci of subcentimeter classic PTC associated with morphologically benign-appearing thyroid tissue (Fig. 1A, B). Cells in both the benign and malignant components expressed TTF1 and thyroglobulin (Fig. 1C, D). The left fallopian tube was uninvolved. Subsequent genetic testing including mutational analysis (ThyGeNEXT®) and miRNA expression profiling (ThyraMIR®, Interpace Diagnostics, Parsippany, NJ) showed a BRAF V600E mutation as well as high-risk levels of miRNA expression in the malignant elements of the ovarian teratoma [5]. Areas of benign thyroid were found to be negative for BRAF on immunohistochemical stain.

This case was discussed at a multidisciplinary tumor board where an ultrasound evaluation of the thyroid was suggested to rule out potential synchronous primary thyroid cancer. Ultrasound was subsequently performed and revealed a 0.6 cm left lobe thyroid nodule with irregular shape and margin with capsular distortion. Other than an elevated level of antithyroglobulin antibody, the patient’s thyroid function was unremarkable. Fine needle aspiration biopsy (FNA-B) of the nodule was positive for PTC (Bethesda Category VI). FNA-B of two left cervical lymph nodes were negative for malignancy. A fluorodeoxyglucose positron emission tomography/computed tomography (FDG-PET/CT) scan demonstrated no uptake. The patient underwent a total thyroidectomy and central compartment neck dissection.

**Fig. 2:**
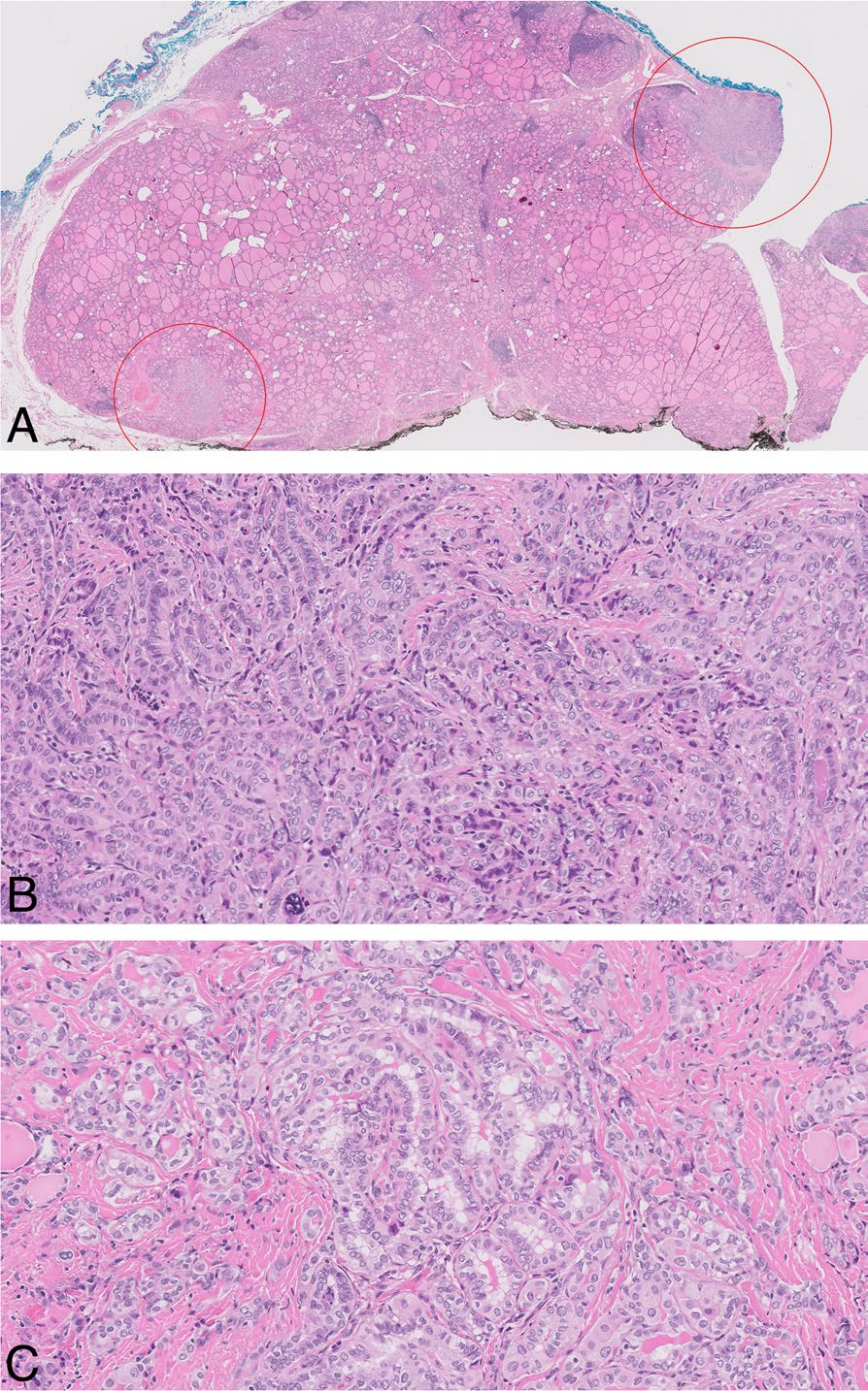
Primary Thyroid Carcinoma. **A** Low power view (0.5x) of H&E section from left thyroid lobe shows two foci of papillary thyroid carcinoma (PTC) present in a background of Hashimoto’s thyroiditis. Focus #1 is indicated by a rectangle and focus #2 is indicated by a circle. **B** High power view (20x) of PTC focus #1. **C** High power view (20x) of PTC focus #2. The neoplastic cells demonstrate characteristic PTC nuclear features (nuclear enlargement, elongation, overlapping, chromatin clearing, irregular nuclear contour, nuclear grooves and nuclear pseudoinclusions). Focus #1 also shows tall cell features with tall cell component estimated 10–20%

**Fig. 3:**
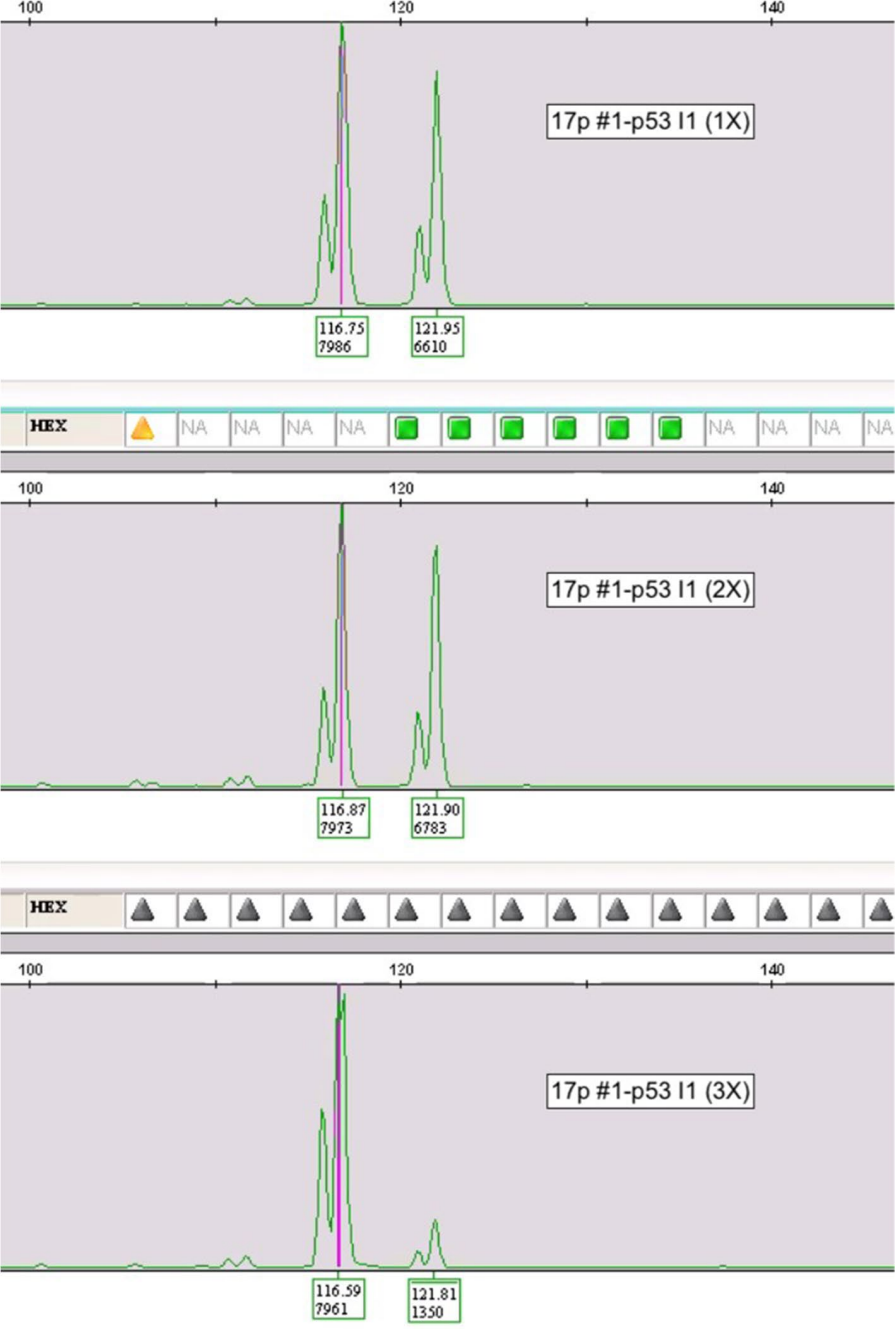
Example of LOH in MSO. TP53 intron 1 pentanucleotide microsatellite. *Top-* non-papillary thyroid cancer struma ovarii target. *Middle-* Thyroid gland microcarcinoma. *Bottom-* Papillary thyroid carcinoma in struma ovarii showing major loss of the larger polymorphic allele

Histopathology demonstrated two foci of subcentimeter PTC of follicular variant measuring 3 mm and 1.5 mm, respectively (Fig. 2A, B, C). Microscopic extrathyroidal extension and tall cell features were present in the 3 mm carcinoma. Lymph nodes were negative. Genetic analysis of each of the two foci revealed BRAF V600E mutation and the same high-risk miRNA expression profile. All tumor deposits were evaluated for TSG loss of heterozygosity (LOH) by assessing allelic imbalance at common loci of known TSG at 1p, 3p, 5q, 9p, 17p, 17q, 18q, 21q and 22q as previously described (example shown in Fig. 3) [6, 7]. No LOH was detected at informative loci in the thyroid PTC. In sharp contrast, LOH was extensively detected in the malignant component of the MSO at 1p, 3p, 5q, 9p, 17p and 22q. The adjacent nonmalignant teratoma tissue showed no LOH.

There were no complications after either surgery. The patient underwent low dose remnant ablation and received 28.3 mCi of iodine-131. One week after treatment, a whole-body diagnostic scan demonstrated no evidence of locoregional or distant metastatic disease. Six months postoperatively, the patient is well with no evidence of recurrent disease on ultrasound.

## Discussion and conclusions

Numerous specific mutations are known important drivers of thyroid cancer. Up to 70% of all PTC have activating mutations in genes that code for signal proteins along the mitogen-activated protein kinase pathway [8]. Approximately 35–40% of all PTC demonstrate BRAF V600E mutations [9–11]. Other common and mutually exclusive driver mutations in PTC include RAS, RET/PTC, BRAF and TERT promoter mutations. Strong driver mutations are highly predictive of malignancy and can be useful in surgical decision making [12–14].

Struma ovarii is an unusual type of mature ovarian teratoma which consists of thyroid tissue [1]. Malignant transformation of the thyroid tissue occurs in approximately 0.5–10% and are termed MSO [15]. Genetic mutations underpin MSO [3, 15–19]. Early studies identified BRAF V600E driver mutations in five of seven case reports of MSO [17, 20]. Characteristic PTC mutations within MSO suggest a common pathogenesis for all PTC, regardless of location. However, limited reported data support this idea.

Synchronous thyroid PTC and MSO are relatively rare, and fewer than 30 patients with synchronous tumors have been identified in recent literature reviews [1, 21]. The mutational status of synchronous tumors have been compared in only seven patients (Table 1). Six patients demonstrated concordant status in MSO and PTC; all lacked BRAF driver mutations, and two patients additionally lacked RET/PTC and KRAS or TERT mutations [16, 21–24]. One patient revealed distinct RAS point mutations in MSO (N-RAS) versus the concurrent PTC (H-RAS) [3]. This is the first report of a patient with concomitant BRAF driver mutations in MSO and PTC. The shared BRAF status suggests a common pathway of carcinogenesis.

**Table 1:** Mutational statuses of synchronous MSO and thyroid PTC cases reported in the literature

Patient Information	Tumor Size(s) *Thyroid PTC*	Tumor Size *MSO*	Molecular *Thyroid PTC*	Molecular *MSO*	Reference
**35 y.o. female**	1.7 cm	8.2 cm*	No BRAF mutation	No BRAF mutation	Capitão et al. (2017) [24]
**62 y.o. female**	0.8 cm	7.2 cm	HRAS Q61R (38% mutant allele freq)	**NRAS Q61R** (50% mutant allele freq)	Gomes-Lima et al. (2018) [3]
**42 y.o. female**	Microcarcinomas, 0.5 cm, 0.8 cm	13.5 cm*	No BRAF, KRAS, or RET/PTC mutation	No BRAF, KRAS, or RET/PTC mutation	Leong et al. (2013) [25]
**47 y.o. female**	0.7 cm	2.7 cm	KIT V530I No BRAF or RAS mutation	KIT V530I No BRAF or RAS mutation	Ma et al. (2016) [16]
**44 y.o. female**	0.05 cm	5 cm*	No BRAF mutation	No BRAF mutation	Marti et al. (2012) [22]
**55 y.o. female**	1.2 cm	1.5 cm	No BRAF mutation	No BRAF mutation	Middelbeek et al. (2017) [23]
**32 y.o. female**	0.6 cm	6.0 cm	No BRAF, RET/PTC, or TERT mutation	No BRAF, RET/PTC, or TERT mutation	Tzelepis (2019) [21]
**42 y.o. female**	0.3 cm, 0.15 cm	3 subcentimeter foci	BRAF V600E mutation High-risk miRNA expression profile	BRAF V600E mutation High-risk miRNA expression profile, **LOH (1p, 3p, 5q, 9p, 17p and 22q)**	Present Case

*Abbreviations: MSO -* Malignant Struma Ovarii,* PTC *- Papillary Thyroid Carcinoma

*Asterisk denotes size of ovary in the absence of PTC size data; Discordant LOH findings emphasized in **bold**

Regarding this patient’s clinical management, the question arose as to whether the thyroid cancer metastasized to the ovary. This was highly unlikely given that metastatic PTC to ovary is exceedingly rare, is typically bilateral, and often occurs in the setting of widespread disease [26]. Moreover, both foci of the thyroid carcinomas were low-risk—unlikely to produce distant metastasis. The reverse scenario, PTC arising from MSO and metastasizing to the thyroid, was also considered. The distinct phenotypes of PTC (classic in MSO of ovary versus follicular variant in the thyroid) suggested independent primaries from each site. Furthermore, normal thyroid within the teratoma, seen here, is considered evidence of a synchronous primary malignancy within the ovary [25, 26]. Nonetheless, further evidence was sought to definitively discriminate multicentric primary PTC versus metastatic MSO.

Molecular evidence was pursued in three areas involving genomic and epigenomic features related to PTC formation. BRAF V600E point mutation was detected in all tumor deposits. Although this suggests a common carcinogenic pathway, it did not provide unequivocal evidence of a single metastatic primary tumor, because BRAF V600E is the most common PTC driver mutation. BRAF mutations are prevalent among PTCs of low oncologic potential [27]. MiRNA expression was then analyzed using a broad panel of growth to promote and suppress miRNA [5]. The epigenomic profile of miRNA was similar in all cancer deposits, which was unsurprising given the BRAF V600E point mutation they shared. This strong driver mutation likely caused equivalent miRNA expression in all tumor locations. Clear difference was noted between TSG LOH in PTC of the thyroid versus MSO. LOH occurs in chromosomes with homozygous gene alterations; it typically confers loss of a portion of the chromosome. This MSO demonstrated LOH in 6 chromosomes at 1p, 3p, 5q, 9p,17p, and 22q of the marker panel, indicating extensive acquired TSG loss. All thyroid carcinomas lacked TSG LOH. This finding argues against the possibility of metastatic PTC from the ovarian teratoma to the thyroid gland. One would expect TSG loss acquired in the MSO to be reliably maintained through clonal expansion and carried in metastasis.

McCarthy et al. suggest that certain subtypes of carcinoma associated with LOH may behave more aggressively than those without LOH [28]. While LOH has not been studied extensively in PTC, it has been associated with aggressive disease and poor prognosis in malignancies such as gliomas and gastric, pancreatic, and breast cancers [7]. Lin et al. reported LOH 17p and 22q in higher grade PTC [29]. Prior studies of LOH in PTC support the idea that loss of TSG are late events correlated with increased aggressiveness [7, 29].

Management of MSO with synchronous PTC has not been addressed in the literature. Even for isolated MSO, there is no treatment consensus nor standardization of care. Given its rarity, evidence is predominantly anecdotal, drawing from case reports or small series. Goffredo et al. analyzed SEER data and found an association between MSO and increased PTC within the thyroid. The authors suggest regular thyroid imaging following MSO diagnosis [4]. Marti et al. analyzed four patients with MSO confined to the ovary [22]. All underwent pelvic surgery, and one underwent total thyroidectomy; the same patient received radioactive iodine after pathology revealed an incidental 0.5 cm PTC with extrathyroidal extension and central lymph node metastasis. The patients were disease-free at a median 9-year follow-up. A literature review of 53 similar MSO patients who underwent various treatments demonstrated a recurrence rate of 7.5% at 25 years. The authors conclude that radical pelvic surgery and prophylactic total thyroidectomy may be reserved for patients with extra-ovarian spread or distant metastases [22]. Similarly, McGill et al. suggest that pelvic surgery alone may be sufficient for most patients with well-differentiated MSO without metastases [30].

Though complete consensus is lacking, most agree that the need for thyroid surgery and/or radioactive iodine therapy depends on the extent of disease and the associated likelihood of recurrence [1, 21, 22]. Recent studies suggest that risk stratification for MSO should be based on pelvic surgical margins, histopathological features, mutational status, and the size of the PTC foci [31]. Most clinicians suggest longitudinal follow-up thyroid imaging after a diagnosis of MSO regardless of disease extent. Our patient was treated with a left salpingo-oophrectomy and total thyroidectomy followed by low-dose radioactive iodine ablation to enhance follow-up and facilitate identification of metastatic disease.

Here we present the first reported case of MSO and synchronous thyroid carcinoma with concordant BRAF V600E status and discordant LOH findings. Our study adds knowledge about genetic alterations in synchronous papillary thyroid cancer. These alterations may aid diagnosis and warrant further study.
